# Health Locus of Control and Health Anxiety in Patients with COVID-19

**DOI:** 10.1192/j.eurpsy.2023.1665

**Published:** 2023-07-19

**Authors:** E. R. Semenova, E. Deshchenko, E. Pervichko, J. Konyukhovskaya

**Affiliations:** 1 Moscow State University named after ‘M. V. Lomonosov’, Moscow, Russian Federation; 2 Psychology, Moscow State University named after ‘M. V. Lomonosov’, Moscow, Russian Federation

## Abstract

**Introduction:**

Perceived sense of control over one’s health contributes to determining health-related behaviors and an individual’s health status. Therefore, it may enhance vulnerability to health anxiety in response to COVID-19 and influence implementation of preventive strategies and adherence to them. Health anxiety may serve as one of the factors that increase the perception of COVID-19 as dangerous and life-threatening. We hypothesized that external health locus of control may demonstrate a connection with higher levels of health anxiety, whereas internal health locus of control may be considered a protective factor alongside some personality traits due to its role in determination of coping strategies.

**Objectives:**

To assess health locus of control (HLC) in patients with COVID-19 and evaluate its connection with the levels of health anxiety.

**Methods:**

The study has involved 62 participants, average age is 23,4±8,2, with 36 of them being diagnosed with COVID-19 one or more times, average age is 24,5±8,9, whereas 26 of them were healthy, average age is 21,8±7,1. The following methods were used: Brief Illness Perception Questionnaire (modified for COVID-19), Perceived Stress Scale, the state scale from the State-Trait Anxiety Inventory, Short Health Anxiety Inventory, Illness- and Treatment-Related Locus of Control Scale, HEXACO-24 Personality Inventory, Self-Government Test.

**Results:**

COVID-19 patients differed from healthy participants by the following parameters: perceived danger of COVID-19 (31,53±9,51 vs 33,92±11,5; p>0,05); perceived stress (28±8,68 vs 26,5±7,3; p>0,05); state anxiety level (23,3±11,1 vs 25,1±12,5; p>0,05); health anxiety (14,3±6,76 vs 13,8±5,7; p>0,05); internal HLC (18,8±3,24 vs 17,8±4,67; p>0,05); external HLC (5,97±1,89 vs 5,81±1,92; p>0,05); extraversion (11,8±3,36 vs 13,10±3,71; p>0,05).

Correlation analysis has revealed mild positive correlations between health anxiety level and both external HLC (0,32; p<0,05) and chance HLC (0,25; p<0,05), mild negative correlation between health anxiety and internal HLC (-0,18; p>0,05). Analysis of COVID-19 related variables found that health anxiety levels were positively correlated with perceived danger of coronavirus disease (0,37; p<0,01), perceived stress (0,59; p<0,001) in the whole sample. Negative correlation was observed between extraversion and health anxiety (-0,49; p<0,05) in the group of COVID-19 patients.

**Conclusions:**

The results obtained in our study demonstrate the connection of the higher levels of health anxiety in COVID-19 patients with more external orientation of HLC. The connection between extraversion and health anxiety is also observed. Our study indicates that participants diagnosed with COVID-19 one or more times tend to have higher health anxiety levels in comparison to healthy participants.

**Disclosure of Interest:**

None Declared

